# Cognitive Impairment in Newly Diagnosed Patients with Multiple Sclerosis: A Systematic Review of Related Molecular Biomarkers and a Meta-Analysis of Associated Demographic and Disease-Related Characteristics

**DOI:** 10.3390/jcm14082630

**Published:** 2025-04-11

**Authors:** Konstantina Stavrogianni, Vasileios Giannopapas, Dimitrios K. Kitsos, Niki Christouli, Vassiliki Smyrni, Athanasios K. Chasiotis, Alexandra Akrivaki, Evangelia-Makrina Dimitriadou, John S. Tzartos, Georgios Tsivgoulis, George P. Paraskevas, Dimitrios Peschos, Konstantinos I. Tsamis, Sotirios Giannopoulos

**Affiliations:** 1 Second Department of Neurology, Attikon University Hospital, National and Kapodistrian University of Athens, 15772 Athens, Greece; stavrogianni.k@gmail.com (K.S.); bgiannopapas@gmail.com (V.G.); dkitsos@icloud.com (D.K.K.); nichristouli@gmail.com (N.C.); b.smyrni@hotmail.com (V.S.); thanosch1@gmail.com (A.K.C.); alexandra.akrivaki@gmail.com (A.A.); evadim93@hotmail.gr (E.-M.D.); jtzartos@gmail.com (J.S.T.); tsivgoulisgiorg@yahoo.gr (G.T.); geoprskvs44@gmail.com (G.P.P.); 2 Department of Physiology, Faculty of Medicine, School of Health Sciences, University of Ioannina, 45110 Ioannina, Greece; dpeschos@uoi.gr (D.P.); ktsamis@uoi.gr (K.I.T.); 3 Department of Physical Therapy, University of West Attica, 12243 Athens, Greece; 4 Laboratory of Neuromuscular and Cardiovascular Study of Motion (LANECASM), University of West Attica, 12243 Athens, Greece

**Keywords:** multiple sclerosis, neuropsychological impairment, processing speed, biomarkers, neurofilament light chain (NfL)

## Abstract

**Background/Objectives**: Neuropsychological impairment (NI) is common in newly diagnosed patients with multiple sclerosis (pwMS). This study has two main objectives; the systematic review aims to describe the relationship between NI and molecular biomarkers in newly diagnosed pwMS, and the meta-analysis aims to explore the relationship between NI, age, disability status, and disease duration in this patient group. **Methods**: We conducted a systematic review, with 20 studies meeting the inclusion criteria. Out of these, 12 studies were included in the meta-analysis. We analyzed three key cognitive measures—the Symbol Digit Modalities Test (SDMT), the Paced Auditory Serial Addition Test (PASAT), and the Selective Reminding Test–long-term storage (SRT-LTS)—in relation to demographic and MS-related characteristics. **Results**: Neurofilament light chain (NfL) levels were consistently associated with NI, especially a slower information processing speed (IPS). Other biomarkers, including chitinase 3-like 1 (CHI3L1), brain-derived neurotrophic factor (BDNF), apolipoprotein E4 allele (APOE4), and vitamin D, also showed promising correlations with NI. A meta-regression analysis of 2380 pwMS indicated a negative association between SDMT score and disability status (*p* = 0.01). No significant associations were found for the PASAT with age, disability status, or disease duration (*p* > 0.05). **Conclusions**: These findings highlight the role of NfL as a biomarker related to NI in newly diagnosed pwMS and the association between IPS and disability status. Further research is needed with more homogeneous samples in terms of the disease duration, along with standardized cognitive assessments and a broader range of biomarkers, to improve our understanding and management of cognitive difficulties in the early stages of MS.

## 1. Introduction

Multiple sclerosis (MS) is a common autoimmune disorder affecting the central nervous system (CNS), characterized by chronic inflammation, leading to the destruction of the myelin sheath surrounding the nerve fibers. MS features periventricular inflammatory lesions and demyelinating plaques in the CNS. Damage to the oligodendrocytes causes demyelination, and as the disease progresses, the axonal damage becomes irreversible [1].

CNS lesions during the early stages of MS can manifest in a range of neurological symptoms and diverse clinical signs, including optic neuritis, spasticity, weakness, sensory deficits, chronic fatigue, bladder and bowel disturbances, neuropathic pain, and neuropsychological impairment (NI) [2,3]. NI is one of the most prevalent symptoms associated with the disease, affecting 34% to 65% of patients with MS (pwMS) [3]. Cognitive weakening primarily impacts information processing speed (IPS) and attention, with secondary effects on learning, memory, speech, executive functioning, and visuospatial abilities [4,5]. This neurocognitive phenotype aligns with “subcortical dementias”, mainly affecting processes reliant on the connections between the cortical areas of the brain and deep gray matter regions [6].

NI has been shown to be associated with the clinical progression and severity of MS [7]. However, due to MS’s multifactorial nature and variable clinical phenotypes, precise classification and description of MS-related NI are challenging, especially during the early phases, on which the research is not systematic and there is fragmented picture of the possible neuropsychological difficulties that these patients face. Nevertheless, NI may occur early in the disease course, even at the time of diagnosis, and may precede physical symptoms and disability, significantly impacting the daily functioning and quality of life (QoL) of pwMS [8]. In this frame, the early detection of cognitive difficulties becomes of prominent importance since it allows for timely interventions and support to mitigate a further decline and its associated consequences.

For MS, various biomarkers derived from the plasma, serum, and cerebrospinal fluid (CSF) have emerged as indicators of neuronal damage, demyelination, and inflammation, all of which are closely tied to the disease’s pathology, clinical presentation, and progression [9]. Despite increasing recognition of their importance and their regular utilization in neurodegenerative diseases such as Alzheimer’s disease (AD), it is noteworthy that no specific soluble biomarker has been identified to reliably predict NI in MS [10], thus obstructing the early detection and prognostication of NI. Notably, certain immunocellular and molecular markers, such as neurofilament light chain (NfL), have been associated with NI in MS. Elevated levels of NfL are linked to axonal damage, exacerbating the neuropsychological profile of the disease [11]. A recent meta-analysis highlighted the potential roles of other molecular markers as well, including brain-derived neurotrophic factor (BDNF), homocysteine, vitamin B12, Tau protein, and various pro- and anti-inflammatory markers, in influencing cognitive function across the trajectory of MS. However, the precise roles of these biomarkers in NI remain incompletely understood, with significant heterogeneity observed across studies [12].

Since the majority of studies have focused on NI primarily during the later phases of MS and on specific types of MS [12], a relatively unexplained region of interest regarding the possible NI during the very early phases of the disease, even at the time of a formal diagnosis, emerges. Some studies have recently initiated systematic explorations and descriptions of the NI profile in newly diagnosed pwMS and the relationship between NI and specific molecular biomarkers. Yet there is an ongoing need to identify NI early in the disease course since it seems to be a significant predictor of the disease’s prognosis [7] and to try to capture key molecular biomarkers for NI in newly diagnosed pwMS, thereby facilitating early prognostication and targeted early interventions.

Building on this, this study has two main objectives. First, our review aims to explore advancements in understanding the role of molecular biomarkers in the cognitive profiles of newly diagnosed pwMS. Second, our meta-analysis seeks to identify the primary associations of NI in this patient group with key demographic and disease-related characteristics.

## 2. Methods

### 2.1. The Standard Protocol and Registration

The protocol for this systematic review and meta-analysis was pre-registered on the Open Search Framework (OSF) platform (https://osf.io/nygmd, accessed on 12 April 2024). This study followed the guidelines outlined in the Preferred Reporting Items for Systematic Reviews and Meta-Analyses (PRISMA) [13] and the Cochrane Handbook for Systematic Reviews [14]. Given the design of this study, ethical approval was not necessary.

### 2.2. The Search Strategy, Selection Criteria, and Process

A systematic literature search was conducted across the databases of PubMed, MEDLINE, Scopus, Science Direct, and the Cochrane Library. The review period spanned from 1 March 2023 to 31 December 2024, with a focus on articles published at any time due to this study’s highly specific focus. Keywords such as “newly diagnosed Multiple Sclerosis patients” and “recently diagnosed Multiple Sclerosis patients” were combined with terms related to biomarkers like “NfL”, “BDNF”, and “biomarkers”, as well as terms associated with cognitive impairment, such as “cognitive decline” and “neuropsychological impairment” (Details on the complete search algorithm are available in the Supplementary Material). Additionally, reference lists of potential included articles and other related systematic reviews and meta-analyses were reviewed to potentially identify relevant articles.

To be included, studies needed to fulfill the following criteria: (1) The inclusion of a population with a recently confirmed diagnosis of MS. Newly diagnosed pwMS were defined, as indicated within the studies, as individuals who had received their diagnosis within a timeframe ranging from less than 6 months to up to 4 years. Some studies also included individuals with clinically isolated syndrome (CIS) in their samples given its potential progression into MS and thus its possible relevance to early-stage research. (2) The inclusion of at least one neuropsychological test. (3) The inclusion of at least one plasma, serum, or CSF biomarker. The exclusion criteria encompassed the following: (1) studies focusing on pwMS without specifying newly diagnosed cases, (2) studies concentrating solely on biomarkers without any cognitive assessment, (3) studies including only neuroimaging parameters without molecular biomarkers, (4) research involving pediatric MS, animal studies, and in vitro analyses, (5) theses lacking full-text papers, case reports, protocols, non-peer reviewed studies, reviews, and meta-analyses, (6) papers inaccessible in full text, and (7) articles not written in English.

Three authors (K.S., N.C., and V.G.) independently carried out the study selection process. Any disagreements were resolved by a third, independent author (S.G.).

### 2.3. Quality Control and Bias Assessment

Two independent authors (K.S., V.G.) evaluated the studies included for their quality and risk of bias (RoB) using the Risk of Bias In Non-randomized Studies—of Interventions (ROBINS-I V2) assessment tool [15]. Any potential disagreements were resolved through a consensus.

The data extracted from the eligible studies included (a) the first author’s name, (b) the year of publication, (c) the study design, (d) the total sample size, (e) the mean scores for the neuropsychological tests of interest [the Symbol Digit Modalities Test (SDMT); the Paced Auditory Serial Addition Test (PASAT); and the Selective Reminder Test–long-term storage (SLR-LTS)], (f) the standard deviations (SDs) for these tests, (g) the mean age of the participants, (h) the mean Expanded Disability Status Scale (EDSS) scores, and (i) the mean disease duration.

### 2.4. Outcomes

A meta-analysis of the aggregate data was performed with the identified studies included. The predefined primary outcomes included the mean scores of the three neuropsychological tests (SDMT, PASAT, and SRT) in newly diagnosed PwMS. The secondary outcomes included potential associations between the primary outcomes and demographic and MS-related characteristics.

### 2.5. Statistical Analysis

The statistical analyses and figure production were conducted using RStudio for Windows (Version 4.4.2) [RStudio/the R Meta package] [16]. The primary outcomes focused on the mean scores from the three neuropsychological tests in newly diagnosed pwMS, while the secondary outcomes explored the associations with demographic and MS-related characteristics. For the aggregated meta-analysis of the pooled mean values for each primary outcome, the meta-mean function from R-Meta was employed.

A random-effect meta-analytical model [17] was used to calculate the pooled estimates and the corresponding 95% confidence intervals (95% CIs). Heterogeneity was assessed using I2 values (>50% and >75% indicating substantial and considerable heterogeneity, respectively) [18]. The statistical significance level for the Q statistic was set to 0.1.

To assess the publication bias across individual studies for the primary outcome, funnel plot inspection, Egger’s linear regression test, and the equivalent z test for each pooled estimate were employed, with a two-tailed *p* value < 0.05 considered statistically significant [19,20].

All of the data generated and analyzed for this study are included within this article and its Supplementary Material Files.

## 3. Results

### 3.1. The Literature Search and Included Studies

**Figure 1:**
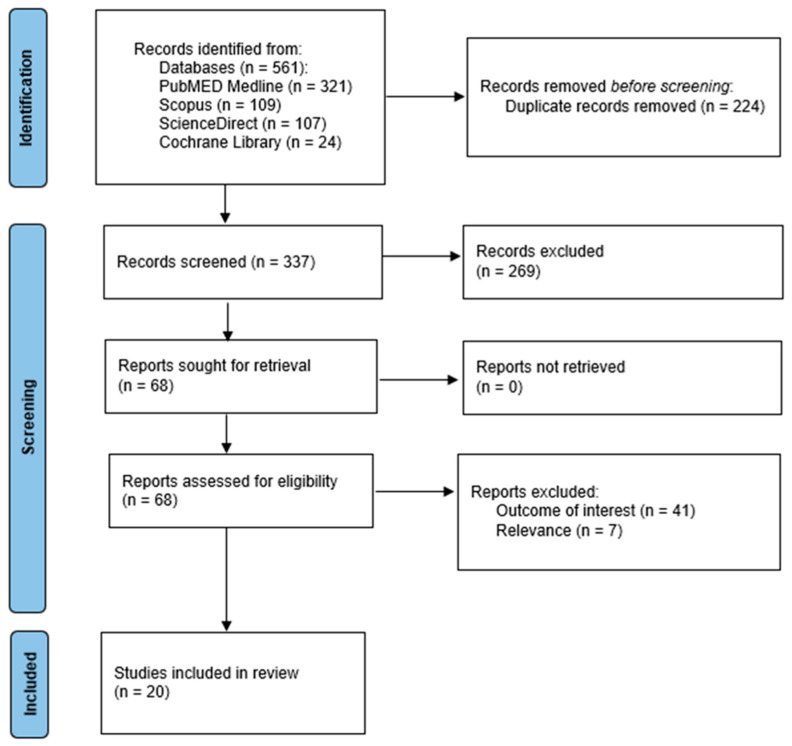
PRISMA flow chart for the study selection.

The initial search yielded a substantial pool of 561 records. After removing duplicates and reviewing the records, 541 records were excluded due to them being out of scope or irrelevant to the systematic review and meta-analysis (Figure 1) [21]. This left 20 records eligible for consideration (Table 1) [7,22,23,24,25,26,27,28,29,30,31,32,33,34,35,36,37,38,39,40].

### 3.2. Quality Control

The RoB was assessed using the ROBINS-I V2 tool. Most of the included studies presented a low RoB, while a significant portion had a moderate RoB. This was primarily due to a lack of consideration of key confounding factors, such as differentiating MS from CIS, and reconsidering core demographic and individual variables like the gender proportions, education level, and IQ scores. Additionally, the absence of a reported blinded process for the neuropsychological and clinical interview assessments contributed to the moderate RoB (Supplementary Figures S1 and S2).

### 3.3. The Qualitative Results of the Systematic Review on Cognitive Performance and Molecular Biomarkers in Newly Diagnosed pwMS

#### 3.3.1. Biomarkers of Axonal Pathology and NI

In total, six studies [23,24,27,33,36,40] have investigated the relationship between a biomarker of axonal pathology, NfL, and NI in newly diagnosed pwMS, with all but one demonstrating significant associations.

Brummer and colleagues [23] found that higher serum NfL (sNfL) levels significantly correlated with worse SDMT scores (*p* = 0.004), indicating an impaired IPS. Cruz-Gomez and colleagues [24] similarly reported elevated sNfL levels, along with an elevated global brain cortical thickness, as significant predictors of worse cognitive outcomes (*p* < 0.001), with pwMS performing worse than healthy controls on the PASAT (*p* = 0.017) and SRT (*p* = 0.009). Gaetani and colleagues [27] observed associations between higher CSF-NfL levels and overall NI (*p* < 0.01), IPS (*p* < 0.05), and semantic verbal fluency (*p* < 0.05) in newly diagnosed pwMS. Quintana and colleagues [36] further supported these findings with correlations between CSF-NfL and verbal fluency, linking elevated biomarker levels to more severe cognitive impairments. Yalachkov and colleagues [40] contributed by showing a negative correlation between cognitive performance and CSF-NfL levels (*p* = 0.032), with higher levels associated with a lower performance across IPS and verbal learning/memory consolidation (*p* = 0.048 and *p* = 0.02, respectively).

Conversely, Pitteri and colleagues [33] presented contradictory findings, reporting no significant correlation between CSF-NfL levels and neuropsychological test scores (*p* > 0.05). These authors suggested that other inflammatory profiles in the CSF, rather than NfL levels alone, may be more relevant to the severity of NI in newly diagnosed pwMS (Table 2).

#### 3.3.2. Biomarkers Modulating Inflammatory Responses and NI

Pitteri and colleagues [33] examined 57 different CSF molecules and their associations with a neuropsychological battery. Their results indicated that the SDMT scores were negatively associated with interleukin 11 (IL11), while the PASAT scores were negatively associated with IL34, chitinase 3-like 1 (CHI3L1), and C-X-C motif chemokine ligand 12 (CXCL12). Additionally, the SLT-LTS scores were negatively associated with C-C motif chemokines [8,13,22] (CCL22, CCL13, CCL8), CXCL10, CXCL12, IL35, macrophage migration inhibitory factor (MIF), and a proliferation-inducing ligand (APRIL) (*p* < 0.05). In a related study with similar results regarding the IPS, Quintana and colleagues [36] explored the link between CSF-CHI3L1 levels and cognitive function. The authors found a negative association between CSF-CHI3L1 levels and performance in Trail Making Test A (*p* = 0.016), suggesting that elevated CSF-CHI3L1 levels may indicate more severe deficits in graphokinetic speed during the early stages of MS (Table 2).

#### 3.3.3. Biomarkers of Neuronal Survival and Metabolism and NI

Only a limited number of studies (n = 4) [26,35,39,40] have investigated different biomarkers that are associated with neuronal survival and metabolism and their potential role in cognitive function in newly diagnosed pwMS, yet their findings collectively suggest that these biomarkers may play a critical role in cognitive function and warrant further investigation in the context of MS.

Engel and colleagues [26] examined the impact of apolipoprotein E (APOE) polymorphisms on cognitive function in CIS/RRMS patients, revealing that APOE4 homozygosity was linked to a reduced overall cognitive performance (*p* = 0.020). Virgilio and colleagues [39] focused on vitamin D levels and found a significantly lower IPS, as measured using the PASAT (*p* = 0.004). Yalachkov and colleagues [40] investigated BDNF and NfL levels, uncovering a negative correlation between cognitive performance and cBDNF (*p* = 0.034), while a composite CSF biomarker that also included NfL showed stronger associations with cognitive outcomes (*p* < 0.05). Lastly, Prokopova and colleagues [35] explored the plasma BDNF concentrations during stress in newly diagnosed pwMS, revealing a lower performance on the Stroop test, which measures executive functioning, compared to that in the healthy controls (*p* < 0.05) (Table 2).

### 3.4. The Quantitative Results of the Meta-Analysis on Cognitive Performance and Its Associations in Newly Diagnosed pwMS

Given the heterogeneity in the types of biomarkers measured and their values, combined with the variability in the cognitive assessment tools used across studies, it was not feasible to conduct a meta-analysis [41]. Therefore, we limited the meta-analysis to three core cognitive measures—the SDMT, PASAT, and SRT-LTS—and their associations with key demographic (age) and MS-related characteristics (disease duration and EDSS score).

#### 3.4.1. Primary Outcomes

A total of 12 studies [22,24,25,27,28,29,31,32,33,38,39,40] involving 2380 newly diagnosed pwMS were included in the meta-analysis. The studies were stratified into three groups based on the reported neuropsychological outcome. Group A included 10 studies reporting the SDMT scores [22,24,25,27,29,31,32,38,39,40] for 1196 pwMS [mean age = 36.08 (SD = 2.3)]. Group B included eight studies reporting the PASAT scores [24,25,27,28,29,32,33,38,39,40] for 1627 pwMS [mean age = 36.2 (SD = 2.3)], and Group C included five studies [24,27,29,32,33] reporting the SRT-LTS scores for 360 pwMS [mean age = 36.82 (SD = 2.8)].

**Figure 2:**
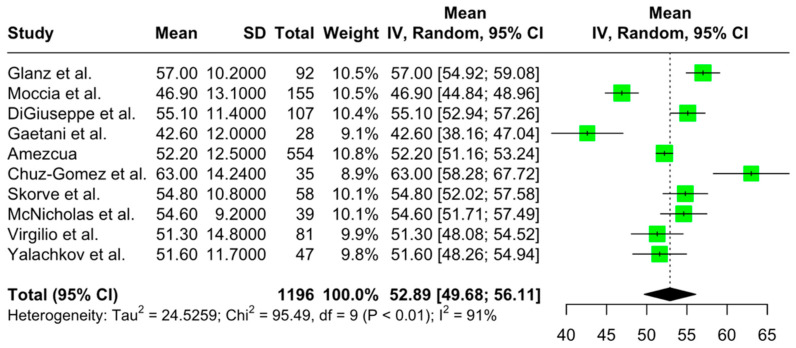
Forest plot of pooled mean SDMT scores [22,24,25,27,29,31,32,38,39,40].

**Figure 3:**
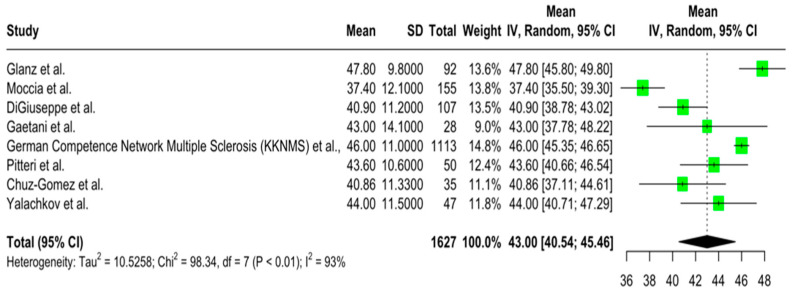
Forest plot of pooled mean PASAT scores [24,25,27,28,29,32,33,40].

**Figure 4:**
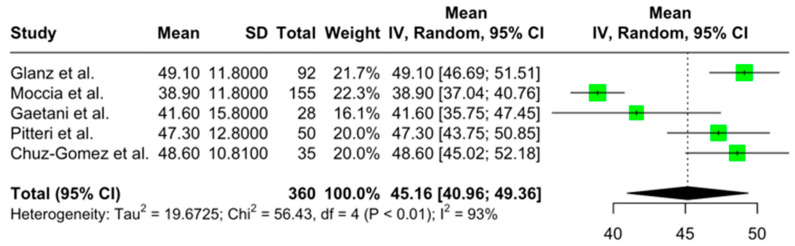
Forest plot of pooled mean memory scores in pwMS [24,27,29,32,33].

The aggregated pooled mean SDMT score was 52.89 (95% CI = [49.68, 56.10]; I2 = 90.6%; *p* < 0.001) (Figure 2). The aggregated pooled mean PASAT score was 43.00 (95% CI = [40.53, 45.46]; I2 = 92.9%; *p* < 0.001) (Figure 3). Finally, the aggregated pooled mean for the SRT-LTS score was 45.15 (95% CI = [40.95, 49.35]; I2 = 92.9%; *p* < 0.001) (Figure 4).

#### 3.4.2. Secondary Outcomes

Potential associations between the aggregated outcomes and the demographic and MS-related characteristics were examined using meta-regression techniques if eight or more studies had reported data on the primary outcome. As such, meta-regression was applied only to the SDMT and PASAT scores.

There was a statistically significant association between the mean SDMT scores and EDSS scores (β = −9.27, *p* = 0.01), which indicated a 9.27 decrease in the SDMT score with every 1.0-point increase in the EDSS. There was no statistically significant association between mean SDMT score and age (*p* = 0.68) or between SDMT score and disease duration (*p* = 0.78). For the PASAT, there was not a statistically significant relationship with the EDSS (*p* = 0.55), age (*p* = 0.46), or disease duration (*p* = 0.86).

#### 3.4.3. Publication Bias

The publication bias was assessed according to funnel plot asymmetry and Egger’s linear test (if at least 10 studies reported outcomes) [42]. There was low evidence of publication bias for the SDMT and low evidence of an effect of a small sample size (Supplementary Figure S3; Egger’s test: β = 0.64, *p* = 0.80) and a medium level of evidence of publication bias for the PASAT (Supplementary Figure S4) and the SRT-LTS (Supplementary Figure S5).

## 4. Discussion

The present systematic review and meta-analysis offers insights into NI and associated biomarkers, age, and key MS-related characteristics in newly diagnosed pwMS. We identified 20 studies that met our inclusion criteria for this systematic review [7,22,23,24,25,26,27,28,29,30,31,32,33,34,35,36,37,38,39,40]. Our review demonstrated that elevated levels of NfL, a marker of axonal damage, were consistently linked to poorer cognitive performance, particularly a poorer IPS. Other biomarkers, such as CSF-CHI3L1, APOE4, vitamin D, and BDNF, also showed some promising significant correlations with the cognitive outcomes. Notably, our meta-analysis revealed a significant association between EDSS and SDMT scores, suggesting a relationship between disability status and cognitive function even at the early stages of MS. In contrast, the PASAT scores were independent of disability progression, age, and disease duration. This suggests that while certain cognitive domains may be influenced by physical disability in MS, NI cannot be solely explained by individual- or disease-specific characteristics. This also emphasizes the necessity of further investigation, particularly focusing on closely related CNS markers that may offer greater insight during the early phases of the disease.

The mechanisms underlying NI onset and progression in MS remain poorly understood. The lesion loads in both the white and gray matter are suggested as determinants of NI, with disruptions in the neuronal circuits significantly contributing to a reduced cognitive performance, especially IPS capacity. These disruptions could potentially be quantified and monitored using biomarkers that complement the standard neuroimaging techniques [27]. A recent review [10] and a meta-analysis [12] have both examined various biomarkers in MS-related NI across the disease’s progression, emphasizing NfL, particularly in the CSF, as a robust indicator of effects on the CNS. Since the NfL protein is a key structural element of myelinated axons and critical to efficient nerve conduction, elevated NfL levels have become an important marker of axonal damage and neurodegeneration in MS [24]. This can explain why subcortical cognitive decline, largely driven by axonal damage, is strongly associated with increased NfL levels in MS [10,12]. Our findings align with these studies, highlighting NfL’s importance and potential diagnostic utility. The research on other potential biomarkers in newly diagnosed pwMS remains limited, limiting our ability to draw definitive conclusions. Thus, our study provides a basic overview of the existing literature on biomarkers, refraining from hypothesizing or making conclusive statements at this stage.

In this study, we chose to present pooled means for the SDMT, PASAT, and SRT-LTS scores without providing a qualitative comparison with the previously established norms for general or MS populations. This decision stemmed primarily from the challenges associated with comparing these scores against normative data from either the general population or from pwMS with longer disease durations. The normative data on these neuropsychological tests in the general population exhibit extensive variability [43], making it difficult to establish meaningful comparisons. Furthermore, the absence of established norms specifically tailored to pwMS or a consensus on the clinical significance of performance in cognitive tests, or changes therein [44], further complicates the interpretation of these scores within our study context. Therefore, even though detailed comparisons were not feasible under these circumstances taking into consideration the already published data regarding information processing time in the MS population, the results of this analysis suggest that even newly diagnosed patients with MS present with signs of cognitive decline.

An important aspect to consider regarding the meta-analytical data, however, is the correlation observed between the SDMT and EDSS scores, which may be attributed to the nature of the SDMT itself. While some studies have used the oral version of the task to minimize the motor component [22,39], the emergence of disability as the sole correlated factor, rather than age or disease duration, may suggest a common brain region supporting both physical disability and IPS, notably the corpus callosum [45]. Although volumetric and MRI data were not within our study’s scope, a previous longitudinal study highlighted that decreases in the volume of the corpus callosum were strongly correlated with both SDMT and EDSS scores [45], suggesting it as a potential mediator of this relationship. The authors reported that during the initial stage of MS, the atrophy in areas with a high axonal content is much more aggressive and extensive and tends to decrease over time with the progression of the disease and the transformation into progression types [45]. This aligns with our finding through meta-regression and may plausibly explain why the SDMT performance remained uncorrelated with age or disease duration, two variables with less variability within these studies and during this stage of the disease. Given that the SDMT primarily measures the IPS, which is supported by the integrity of the white matter [46], extensive lesions and inflammatory processes affecting connectivity could significantly impact its performance.

The PASAT appeared to be uncorrelated with all of the demographic and MS-related characteristics. As a neuropsychological measure, the PASAT involves multiple cognitive functions beyond IPS, including auditory processing, mathematical abilities, working memory, attention, and concentration; thus, it cannot be seen as a unitary concept [47]. Matias-Guiu and colleagues [47] found that the performance in the PASAT in pwMS was linked to the status of various brain regions, such as the posterior cingulate cortex/precuneus, the basal ganglia, and the cerebellum. These regions are likely involved in fronto-subcortical and default mode networks. Conversely, while white matter lesions may contribute to performance, specific localizations associated with performance in the PASAT were not possible to identify. The authors concluded that the PASAT might measure the efficiency of cognitive effort and concentration in high-demand attentional tasks, reflecting the preservation of both cortical and subcortical structures [47]. As such, given that the PASAT is supported by multiple brain circuits, deficits in one area may be compensated for by the intact functioning of other areas. This complexity and the involvement of multiple networks might explain why performance in the PASAT remained independent of factors like age, disease duration, and disability progression during the early stages of the disease, where overall cognitive function had not yet been notably impaired.

The studies included in this review utilized a series of heterogeneous cognitive batteries and tests to assess NI, making direct comparisons challenging. The Brief Repeatable Battery of Neuropsychological Tests (BRB-N) emerged as the most frequently used battery [24,27,29,32,33,34,36,37], with the SDMT and PASAT being the most commonly used individual tasks [7,22,23,26,28,39,40]. Beyond these, the inclusion of a diverse variety of verbal memory tests, and to a lesser extent, spatial memory tests introduced significant variability, complicating any conclusions regarding this cognitive component. Additionally, other critical cognitive domains such as executive functions (e.g., inhibition, working memory, cognitive flexibility, planning, problem-solving, abstract reasoning) and language-related components (e.g., fluency, naming, writing) have largely been overlooked in most studies. It is important to note that caution should be exercised when assessing these latter domains because many tests rely heavily on restricted completion times [48], which could artificially inflate any deficits. It is highly recommended that future studies consider including tasks or measures within tasks that are not time-dependent or account for the potential impact of deteriorations in IPS on the results.

Upon the initial revelation of an MS diagnosis, patients encounter various stressors, including the diagnosis itself, the unpredictability of the disease, symptoms, relapses, and consequential life adjustments. Research indicates that individuals recently diagnosed with MS often experience heightened levels of psycho-emotional distress [49]. Stress is known to impact the endocrine system, leading to changes in brain connectivity between emotional regulation centers like the limbic system (including the hippocampus and the amygdala) and regions involved in higher cognitive functions such as the prefrontal cortex and the orbitofrontal cortex. These brain circuits traditionally regulate executive processes, fluency, and selective memory [50]. Given the elevated anxiety symptoms that patients may experience during the early phase of diagnosis, it is important to systematically assess not only fundamental cognitive functions like IPS, attention, and memory—typically known to be affected in MS—but also more complex cognitive domains, that may be more susceptible to alterations due to microstructural changes and vulnerabilities in microconnections.

A notable observation is that while many studies claim to have included newly diagnosed pwMS, there is a scarcity of research specifically focused on individuals within a close timeframe to the initial diagnosis (e.g., at diagnosis or within a few weeks) [39]. Furthermore, there is a lack of a consensus regarding what constitutes “newly diagnosed” pwMS in the literature. Consequently, most studies tend to encompass patients across a broad range of timeframes—from several months to up to four years post-diagnosis—often including pwMS who have already initiated therapy and may have mild to moderate disability levels [40]. This extended and heterogeneous timeframe can complicate the findings, as the cognitive profiles and biomarker levels can vary significantly with the disease progression and treatment effects [51]. Early-stage MS may present unique neuropsychological characteristics that could be diluted or altered over time. Therefore, there is an urgent need for more targeted research specifically examining the cognitive and biomarker profiles at the time of diagnosis or shortly thereafter.

A final noteworthy observation is that longitudinal studies on NI in MS have documented improvements across several cognitive domains in subsequent assessments. This shift may be attributed to several factors. Firstly, practice effects could occur due to repeated testing using the same measures during follow-up examinations. Secondly, as patients become accustomed to their MS diagnosis and less anxious about their disease progression over time, this might lead to better scores on the self-reported psychological scales, potentially influencing cognitive performance. Additionally, the administration of disease-modifying therapies (DMTs) might also contribute to these improvements [7]. To deepen our understanding, it is of high importance to conduct more longitudinal studies that systematically consider these factors. Furthermore, efforts should focus on standardizing the variations and versions of established neuropsychological batteries and tasks sensitive to MS pathology. This standardization could mitigate potential biases from practice effects and ensure more precise evaluations of cognitive function over time.

### Limitations and Future Directions

This study represents the first attempt in the literature to estimate and correlate NI with demographic- and disease-related characteristics while also reviewing the field’s current understanding of the relationship between NI and molecular biomarkers in newly diagnosed pwMS. However, several limitations need acknowledgment. Firstly, the sample size is relatively small for yielding definitive conclusions on newly diagnosed pwMS. Secondly, the lack of a specified criterion for defining “newly diagnosed” in MS has contributed to a sample with significant variability in the time elapsed since a diagnosis at the time that cognitive assessments were conducted. Thirdly, the inclusion of patients with CIS, a condition closely related to MS but with a lower frequency and intensity of NI compared to those of definite MS [52], could limit the power of the results due to this combined profile. Additionally, other potentially relevant factors beyond age, disease duration, and disability status, such as the presence of fatigue, self-reported anxiety and depression, therapy duration, and treatment status (naïve pwMS or those under treatment), could not be included in the meta-regression analysis due to their inconsistent measurement. Finally, due to the omitted data regarding the differences between pwMS without cognitive problems and those with mild, moderate, or severe NI, the results of this study must be interpreted cautiously.

The current state of the research on the cognitive profile of newly diagnosed pwMS is hindered by methodological limitations, leading to gaps in our understanding. To address this, future studies should focus on systematic approaches to studying new diagnoses; emphasizing larger, more homogenous samples; comprehensive assessments of cognition; longitudinal designs ideally starting before the initiation of treatment; the incorporation of molecular biomarkers; the consideration of diverse influencing factors; and standardization of the measurement protocols.

## 5. Conclusions

This study reviewed 20 research articles focusing on NI in newly diagnosed pwMS. Among these, 9 explored the potential links between NI and some molecular biomarkers, while 12 were included in a meta-analysis examining the correlations between NI and age, disability status, and disease duration. The systematic review showed that elevated NfL levels were consistently correlated with a poorer cognitive performance, particularly in IPS. Other biomarkers like CSF-CHI3L1, APOE4, vitamin D, and BDNF also seem to be associated with cognitive outcomes. The meta-analytical results indicated a significant association between EDSS and SDMT scores, highlighting the impact of disability status on cognition in early MS. However, the PASAT scores were independent of demographic and disease-related characteristics. This study emphasizes the critical need for more targeted research on the cognitive and biomarker profiles of newly diagnosed pwMS shortly after diagnosis. Future investigations should prioritize larger, more homogeneous samples and longitudinal study designs to deepen our understanding and refine the clinical management strategies during this critical stage of MS. Given the increased heterogeneity and the evidence of a moderate level of publication bias, especially as regards the PASAT and SLR-LTS, there is a need for structured, standardized protocols regarding the neuropsychological assessment of newly diagnosed patients in order to establish an accurate baseline.

## Figures and Tables

**Table 1:** Characteristics of the studies in the systematic review.

Author	Year	Region	Study Design	Sample Size	MS/CIS	Disease Duration ^1^	Male/ Female
Amezcua et al. [22]	2020	USA	Cross-sectional	554		<1	160/394
Brummer et al. [23]	2022	Germany	Cross-sectional	152	118/34	1.4	45/107
Cruz-Gomez et al. [24]	2020	Spain	Cross-sectional	35	35	3	20/15
DiGiuseppe et al. [25]	2018	England	Cross-sectional	107	107	<1	25/82
Engel et al. [26]	2020	Germany	Cross-sectional	552	308/244	4	157/395
Gaetani et al. [27]	2019	Italy	Cross-sectional	28	25/3	2.5	8/20
German Competence Network of Multiple Sclerosis et al. [28]	2019	Germany	Longitudinal	1113	622/501	<1	338/775
Glanz et al. [29]	2007	USA	Cross-sectional	92	77/15	<1	21/71
Hankomäki et al. [30]	2014	Finland	Longitudinal	36	36		12/24
Jønsson et al. [7]	2006	Denmark	Longitudinal	80	80	<1	
McNicholas et al. [31]	2021	Ireland	Cross-sectional	39	39	<1	
Moccia et al. [32]	2016	Italy	Longitudinal	155	155	<1	56/99
Pitteri et al. [33]	2022	Italy	Cross-sectional	69	64/5	3.4	15/54
Pitteri et al. [34]	2019	Italy	Cross-sectional	50	50	3.5	13/37
Prokopova et al. [35]	2017	Slovakia	Cross-sectional	19	19		9/10
Quintana et al. [36]	2018	Spain	Cross-sectional	51	51		12/39
Ruet et al. [37]	2013	France	Longitudinal	69	69	2.6	24/45
Skorve et al. [38]	2020	Norway	Longitudinal	58	58	1.2	14/44
Virgilio et al. [39]	2021	Switzerland	Cross-sectional	81	79/2	<1	27/54
Yalachkov et al. [40]	2022	Germany	Cross-sectional	47	38/9		12/34

Notes. MS: multiple sclerosis, CIS: clinically isolated syndrome. ^1^ Disease duration is reported in years.

**Table 2:** Characteristics of the study sample and measures included in the systematic review.

Author	Mean Age	EDSS	Neuropsychological Assessment	Psychological Assessment	Biomarker	Male/Female
Amezcua et al. [22]	40.3		SDMT			160/394
Brummer et al. [23]	33.0	1.3	SDMT, PASAT, and VLMT	HADS	sNfL	45/107
Cruz-Gomez et al. [24]	38.4	1	BRB-N	STAI, BDI	sNfL	20/15
DiGiuseppe et al. [25]	35.8	1.8	MACFIMS	HADS		25/82
Engel et al. [26]	32	1.5	MUSIC, PASAT-3	BDI-II	APOE e4	157/395
Gaetani et al. [27]	39.1	2	BRB-N		CSF-NfL	8/20
German Competence Network of Multiple Sclerosis et al. [28]	34.1	1.5	MUSIC, PASAT-3	BDI-II		338/775
Glanz et al. [29]	36.5	1.3	BRB-N	CES-D		21/71
Hankomäki et al. [30]	36		CS, PASAT, SDMT, dual task, WMS-R Logical Memory, SRT, Benton VRT, WAIS-R Similarities and Block Design, VF	BDI-II		12/24
Jønsson et al. [7]	35	2.7	WAIS Vocabulary and Similarities, SSST, Digits Forward and Backward, Design Fluency, SCNT, RPM A-B, SDMT, RCFT, ToL, SRT, Animals, MCT, SGCT, BN, and Famous Faces			
McNicholas et al. [31]	35.3	0.8	PDQ, BICAMS	HADS		
Moccia et al. [32]	32.1	1.8	BRB-N			56/99
Pitteri et al. [33]	37.3	2.0	BRB-N, Stroop test, Phonological, Semantic, and Alternate VF, MFPT	DASS-21	Inflammatory mediators, such as IL11, IL34, IL35, CHI3L1, CXCL12, CCL22, CCL13, CCL8, CXCL10, CXCL12, MIF, APRIL, and CSF-NfL	15/54
Pitteri et al. [34]	38.2	1.5	BRB-N, Stroop test, Phonological, Semantic, Alternate VF, MFPT	DASS-21		13/37
Prokopova et al. [35]	30.6	1.1	Stroop test	STAI, BDI, 8SQ	P cortisol, copeptin, aldosterone, BDNF	9/10
Quintana et al. [36]	35.7	2	BRB-N, TMT-A	HADS-A	CHI3L1, NfL	12/39
Ruet et al. [37]	39.0	2.0	The WAIS-R Similarities subtest, BRB-N			24/45
Skorve et al. [38]	37.6	1.4	BICAMS	HADS		14/44
Virgilio et al. [39]	37.6		SDMT		Vitamin D	27/54
Yalachkov et al. [40]	35.4	2.1	SDMT, PASAT-3, RCFT, VLMT	BDI-II	sNfL, CSF-NfL, sBDNF, CSF-BDNF	12/34

Notes. 8SQ: Eight State Questionnaire; APOE e4: apolipoprotein epsilon 4 polymorphism; APRIL: a-proliferation-inducing ligand; Benton VRT: Benton Visual Retention Test; BDI: Beck Depression Inventory; BDNF: brain-derived neurotrophic factor; BICAMS: Brief International Cognitive Assessment for Multiple Sclerosis; BN: Boston Naming; BRB-N: Brief Repeatable Battery of Neuropsychological Tests; CCLs: C-C motif chemokines; CES-D: Center for Epidemiological Studies Depression Scale; CHI3L1: chitinase-3-like protein 1; CS: CogniSpeed; CSF: cerebrospinal fluid; CXCL: C-X-C motif chemokine ligand; DASS-21: Depression Anxiety Stress Scale; EDSS: Expanded Disability Status Scale; HADS: Hospital Anxiety and Depression Scale; IL: interleukin; MACFIMS: Minimal Assessment of Cognitive Function in Multiple Sclerosis; MCT: Mesulam Cancellation Test; MFPT: Modified Five-Point Test; MIF: macrophage migration inhibitory factor; MUSIC: Multiple Sclerosis Inventarium Cognition; NfL: neurofilament light chain; p: plasma; PASAT: Paced Auditory Serial Addition Test; RPM: Raven Progressive Matrices; PDQ: Perceived Deficits Questionnaire; s: serum; SDMT: Symbol Digit Modalities Test; SGCT: Street Gestalt Completion Test; SSST: Serial Seven Subtraction Test; RCFT: Rey Complex Figure Test; SCNT: Stroop color-naming test; SRT: Spatial Reminder test; STAI: State-Trait Anxiety Inventory; TMT: Trail Marking Test; ToL: Tower of London; VF: Verbal Fluency; VLMT: Verbaler Lern- und Merkfahigkeitstest; WAIS: Wechsler Adult Intelligence Scale; WMS-R: Wechsler Memory Scale, Revised.

## Data Availability

The data presented in this study are available upon request from S.G. (the corresponding author).

## Supplementary Materials

The following supporting information can be downloaded at: https://www.mdpi.com/article/10.3390/jcm14082630/s1, Figure S1: Traffic light plot ROBINS-I tool. Figure S2: Summary of Bias Analysis using the ROBINS-I tool. Figure S3: Funnel plot for pooled mean SDMT scores. Figure S4: Funnel plot for pooled mean PASAT scores. Figure S5: Funnel plot for pooled mean SRT-LTS scores.
