# Supplementary material for: Cognitive Impairment in Newly Diagnosed Patients with Multiple Sclerosis: A Systematic Review of Related Molecular Biomarkers and a Meta-Analysis of Associated Demographic and Disease-Related Characteristics
Source: J Clin Med. 2025 Apr 11;14(8):2630. doi: 10.3390/jcm14082630 (PMC12027780; doi:10.3390/jcm14082630)

Supplementary materials

No of Tables: 0

No of Figures: 5

Figures Legend

Supplementary Figure S1 (SF1): Traffic light plot ROBINS-I tool

Supplementary Figure S2 (SF2): Summary of Bias Analysis using the ROBINS-I tool

Supplementary Figure S3 (SF3): Funnel plot for pooled mean SDMT scores

Supplementary Figure S4 (SF4): Funnel plot for pooled mean PASAT scores

Supplementary Figure S5 (SF5): Funnel plot for pooled mean SRT- LTS scores

Pico Criteria

P: Adults with a new/recent diagnosis of Multiple Sclerosis

I: N/A

C: N/A

O: neuropsychological impairment

Search algorithm

PubMED MEDINE

(newly AND diagnosed AND multiple AND sclerosis AND patients) AND ((cognitive AND impairment) OR (neuropsychological AND decline) OR (cognitive AND decline) OR (cognitive AND deficit) OR (nfl) OR (biomarkers) OR (bdnf))

Science direct and Scopus

(newly AND diagnosed AND multiple AND sclerosis AND patients [Title/Abstract]) AND ((cognitive AND impairment [Title/Abstract]) OR (neuropsychological AND decline [Title/Abstract]) OR (cognitive AND decline [Title/Abstract]) OR (cognitive AND deficit [Title/Abstract]) OR (nfl [Title/Abstract]) OR (stress AND biomarkers [Title/Abstract]) OR (bdnf [Title/Abstract]))

(recently AND diagnosed AND multiple AND sclerosis AND patients [Title/Abstract]) AND ((cognitive AND impairment [Title/Abstract]) OR (neuropsychological AND decline [Title/Abstract]) OR (cognitive AND decline [Title/Abstract]) OR (cognitive AND deficit [Title/Abstract]) OR (nfl [Title/Abstract]) OR (stress AND biomarkers [Title/Abstract]) OR (bdnf [Title/Abstract]))

Cochrane library

(newly AND diagnosed AND multiple AND sclerosis AND patients) AND ((cognitive AND impairment) OR (neuropsychological AND decline) OR (cognitive AND decline) OR (cognitive AND deficit) OR (nfl) OR (stress AND biomarkers) OR (bdnf))

Figure S1

Risk of bias domains

Study	D1	D2	D3	D4	D5	D6	D7	Overall
Amezcua et al. (2020)	-	-	+	+	+	-	+	-
Brummer et al. (2022)	-	-	+	+	+	-	+	-
Cruz-Gomez et al. (2021)	-	+	+	+	+	-	+	+
DiGiuseppe et al. (2018)	-	-	+	+	+	+	+	+
Engel et al. (2020)	-	-	+	+	+	+	+	+
Gaetani et al. (2019)	-	+	-	+	+	+	+	+
German Competence Network Multiple Sclerosis et al. (2019)	-	+	+	+	+	+	+	+
Glanz et al. (2007)	-	+	-	+	+	-	+	-
Hankomäki et al. (2014)	-	+	-	+	-	-	+	-
Jønsson et al. (2006)	-	+	-	+	+	-	+	-
McNicholas et al. (2021)	+	-	-	+	+	+	+	+
Moccia et al. (2016)	-	-	+	+	+	+	+	+
Pitteri et al. (2022)	+	+	+	+	+	-	+	+
Pitteri et al. (2019)	+	+	+	+	+	-	+	+
Prokopova et al. (2017)	-	+	+	+	+	-	+	+
Quintana et al. (2018)	-	?	-	+	+	+	+	-
Ruet et al. (2013)	+	+	+	+	+	+	+	+
Skorve et al. (2020)	-	-	+	+	-	+	+	-
Virgilio et al. (2021)	-	-	+	+	-	+	+	-
Yalachkov et al. (2022)	+	+	+	+	+	+	+	+

Domains:
D1: Bias due to confounding.
D2: Bias due to selection of participants.
D3: Bias in classification of interventions.
D4: Bias due to deviations from intended interventions.
D5: Bias due to missing data.
D6: Bias in measurement of outcomes.
D7: Bias in selection of the reported result.

Judgement
- Moderate
+ Low
? No information

Figure S2

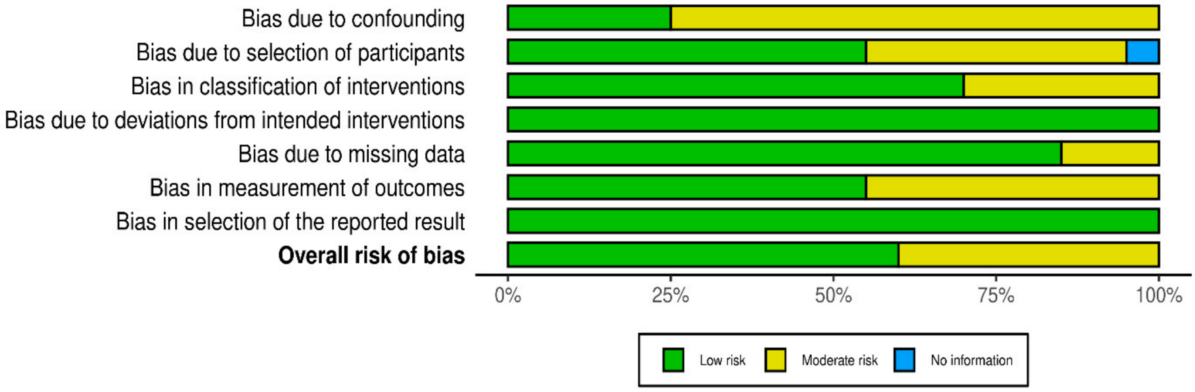

Figure S3

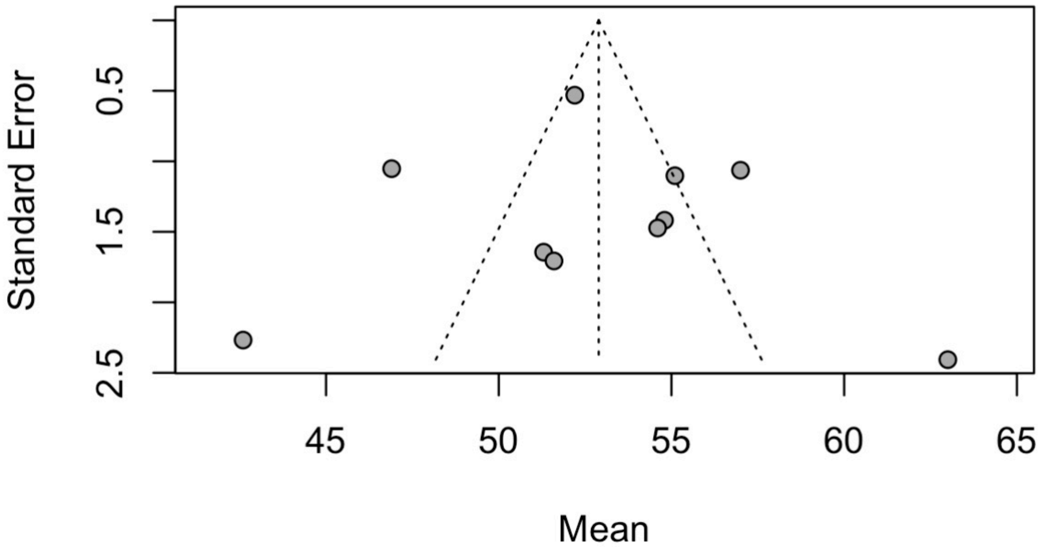

Figure S4

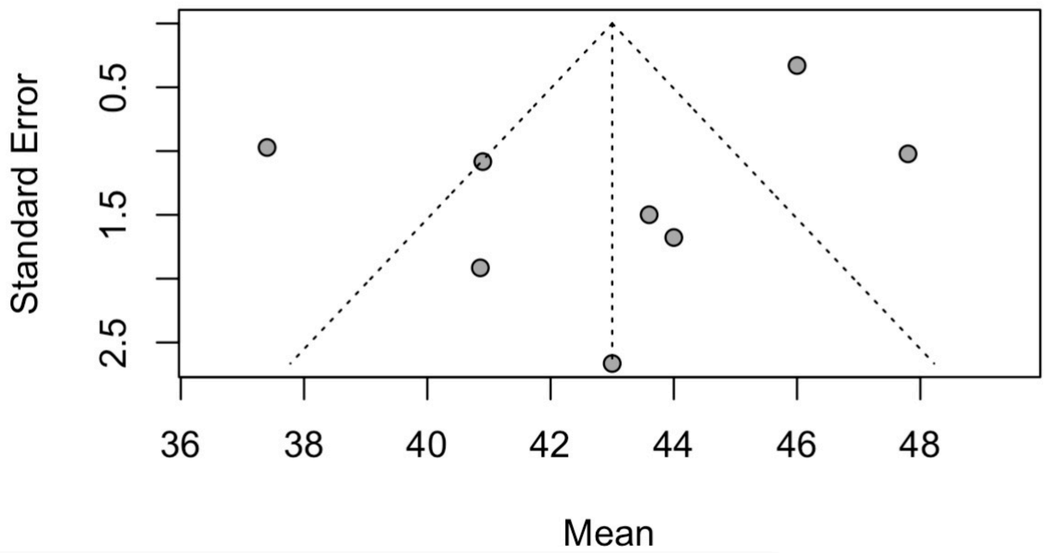

Figure S5

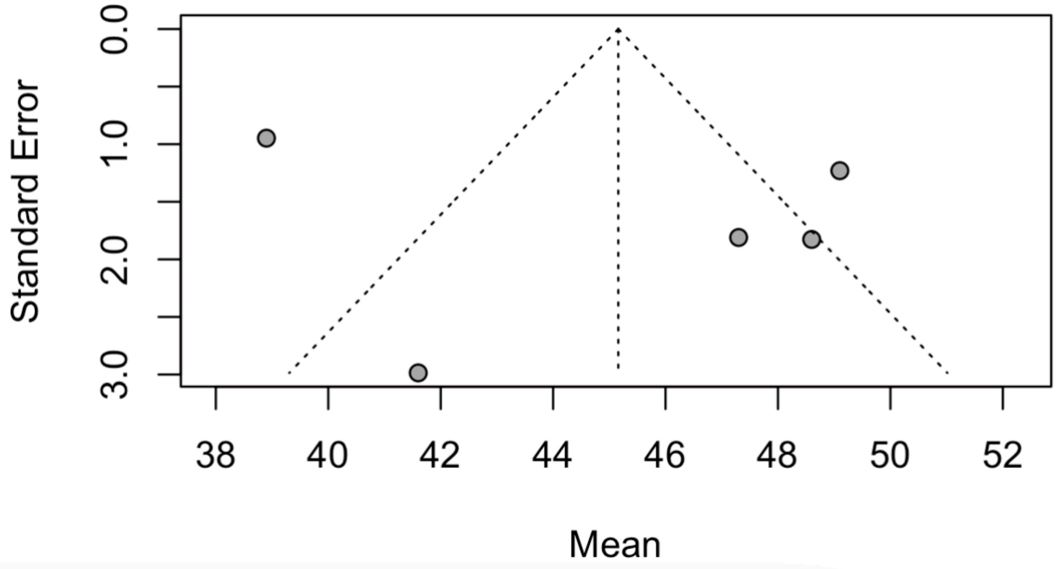

Supplement: Supplementary file 1 [file jcm-14-02630-s001.zip › jcm-3531967-supplementary.pdf]
